# Can specific virtual reality combined with conventional rehabilitation improve poststroke hand motor function? A randomized clinical trial

**DOI:** 10.1186/s12984-023-01170-3

**Published:** 2023-04-04

**Authors:** Marta Rodríguez-Hernández, Begoña Polonio-López, Ana-Isabel Corregidor-Sánchez, José L. Martín-Conty, Alicia Mohedano-Moriano, Juan-José Criado-Álvarez

**Affiliations:** 1 Faculty of Health Sciences, University of Castilla-La Mancha, 45600 Talavera de la Reina, Spain; 2 Technological Innovation Applied to Health Research Group (ITAS Group), Faculty of Health Sciences, University of Castilla-La Mancha, Talavera de la Reina, Spain; 3 Institute of Health Sciences, Talavera de la Reina, Spain

**Keywords:** Randomized controlled trial, Stroke, Neurorehabilitation, Motor recovery, Hand, Virtual reality-based therapy

## Abstract

**Trial objective:**

To verify whether conventional rehabilitation combined with specific virtual reality is more effective than conventional therapy alone in restoring hand motor function and muscle tone after stroke.

**Trial design:**

This prospective single-blind randomized controlled trial compared conventional rehabilitation based on physiotherapy and occupational therapy (control group) with the combination of conventional rehabilitation and specific virtual reality technology (experimental group). Participants were allocated to these groups in a ratio of 1:1. The conventional rehabilitation therapists were blinded to the study, but neither the participants nor the therapist who applied the virtual reality–based therapy could be blinded to the intervention.

**Participants:**

Forty-six patients (43 of whom completed the intervention period and follow-up evaluation) were recruited from the Neurology and Rehabilitation units of the Hospital General Universitario of Talavera de la Reina, Spain.

**Intervention:**

Each participant completed 15 treatment sessions lasting 150 min/session; the sessions took place five consecutive days/week over the course of three weeks. The experimental group received conventional upper-limb strength and motor training (100 min/session) combined with specific virtual reality technology devices (50 min/session); the control group received only conventional training (150 min/session).

**Results:**

As measured by the Ashworth Scale, a decrease in wrist muscle tone was observed in both groups (control and experimental), with a notably larger decrease in the experimental group (baseline mean/postintervention mean: 1.22/0.39; difference between baseline and follow-up: 0.78; 95% confidence interval: 0.38–1.18; effect size = 0.206). Fugl-Meyer Assessment scores were observed to increase in both groups, with a notably larger increase in the experimental group (total motor function: effect size = 0.300; mean: − 35.5; 95% confidence interval: − 38.9 to − 32.0; wrist: effect size = 0.290; mean: − 5.6; 95% confidence interval: − 6.4 to − 4.8; hand: effect size = 0.299; mean: − -8.9; 95% confidence interval: − 10.1 to − 7.6). On the Action Research Arm Test, the experimental group quadrupled its score after the combined intervention (effect size = 0.321; mean: − 32.8; 95% confidence interval: − 40.1 to − 25.5).

**Conclusion:**

The outcomes of the study suggest that conventional rehabilitation combined with a specific virtual reality technology system can be more effective than conventional programs alone in improving hand motor function and voluntary movement and in normalizing muscle tone in subacute stroke patients. With combined treatment, hand and wrist functionality and motion increase; resistance to movement (spasticity) decreases and remains at a reduced level.

*Trials Registry*: International Clinical Trials Registry Platform: ISRCTN27760662 (15/06/2020; retrospectively registered).

## Introduction

Stroke is a leading cause of long-lasting disability. As many as 41.5 million new cases occur yearly in Europe, and 3.7 million survivors experience long-lasting impairments, whereas less than 15% of patients achieve full poststroke recovery [1].

It is estimated that 80% of stroke patients have upper-limb deficits and have decreased activity and use of the paretic hand in daily life [2]; the involvement of the more affected hand in activities of daily living (ADLs) depends on the severity of the deterioration and is associated with a decrease in health-related quality of life (HRQoL) and restrictions on social participation [3, 4].

Most of the functional recovery after diagnosis occurs in the first three months, although neural repair processes and behavioral improvements continue to show slight plasticity in later phases of the rehabilitation process [5, 6]. Therefore, it is crucial that hand rehabilitation begin early; treatment should start within this window of opportunity for functional recovery, when the brain is especially receptive to sensorimotor interaction [7–9].

Rehabilitative treatment of the upper limb is recognized by consensus among survivors, caregivers and health professionals as one of the top ten research priorities for poststroke recovery [10, 11]. In addition to the rehabilitation of the upper limb, other priorities should also be taken into account for the development of neurorehabilitation programs and the design of the corresponding studies, such as minimizing patients’ mobility disability, poststroke fatigue and difficulty in fulfilling responsibilities in the family and work environments; improving patients’ response to the demands of society; and ensuring exhaustive, well-structured monitoring of their clinical evolution after treatment.

During poststroke hand treatment, special attention must be paid to restoring the different biomechanical movements and curvature of the hand in order to provide a stable base and correct alignment as a prerequisite for dexterity training and modulation of reaching movements [12–14]. It is crucial to remember that restoring the selective voluntary movements of the upper limb in stroke patients also relies on the postural control that is necessary for reaching movements—scapula stabilization, shoulder stability more broadly, and selective muscle recruitment [15–20].

Various therapies based on a conventional approach have been demonstrated to be useful, achieving good results in terms of hand rehabilitation: motor imagery training seems to improve the precision and accuracy of movement, as well as the reception of sensory signals, by fostering activation of dormant synapses and accelerating reperfusion of the ischemic penumbra [21]. Mirror therapy can reduce asymmetric hemisphere activation, stimulate the primary motor cortex in both the lesioned (ipsilateral) hemisphere and the opposite (contralateral) hemisphere, widely activate the mirror neuron system and induce partial pathways for motor neurons on the side affected by stroke, which facilitates the remodeling of brain function [22, 23]. Constraint-induced movement therapy focuses on intensive, gradual training of the paretic upper limb to improve its use in specific tasks, limit the use of the less affected upper limb, and, in the context of behavior-changing methods for improving adherence, transfer the clinical achievements into the patient’s real life [24] by relating the therapeutic intervention components to the improvement of motor function and the use and skill of the paretic hand in daily life [25]. Forced use, which is meant to maximize daily use of the paretic hand, seems to yield improvements in motor function after intervention, and these improvements persist for three months after poststroke intervention [26]. Last but not least, active sensory therapies focus on active sensory training in the context of practice with goals involving multiple areas of the brain; pursuing neural reorganization in this manner enhances the motor recovery of the paretic upper limb (e.g., practicing nonvisual identification of common objects increases stereognosis) [27].

Another important aspect of hand-focused therapy programs is the use of a generous dose of intense repetition [28]. Lang et al. [27] determined by means of meta-regression that from 24 to 57 h, the effect size increased by 0.034 for every ten extra hours of therapy, independent of the specific poststroke intervention. In a conventional therapy session at an ordinary hospital rehabilitation unit, a patient can achieve 30 repetitions of an exercise involving the upper limbs, whereas specific technological systems allow more than 300 repetitions in 34 min of action per session [29, 30].

Recent years have witnessed an increased use of technology-based and especially virtual reality–based neurorehabilitation approaches, which have allowed the creation of effective simulated environments and provided multimodal, controllable and customizable stimulation [31]. The re-creation of objects in virtual form maximizes visual feedback [32]. In addition, high intensity and a large number of repetitions are key factors influencing neuroplasticity and functional improvement in patients [33]. Rehabilitation based on virtual reality offers the possibility of addressing individual treatment needs and simultaneously standardizing evaluation and training protocols [34, 35].

There are two major types of virtual reality-based systems used in neurorehabilitation: nonspecific virtual reality (N-SVR) systems and specific virtual reality (SVR) systems. These two classes differ in that systems of the former type use game consoles and video games designed by the entertainment industry. Such consoles (Wii, Xbox, PlayStation, etc.) run games that are not designed for adults suffering from a neurological pathology and do not allow monitoring of movement or other motor or functional variables of the affected body segments. Thus, N-SVR systems are not designed for the neurophysiological recovery of the brain, and they do not focus on the neuronal connections necessary for the recovery of hand function after stroke. In contrast, SVR systems are specifically designed to promote motor learning and recovery, optimizing the acquisition, retention and generalization of motor skills. SVR systems incorporate key features of virtual reality and add objective, quantitative movement monitoring and exergames to facilitate the motor recovery of the hand (regular voluntary movement, arches of hand curvature, grasping, pinch grips and gross manipulation). In addition, SVR systems comply with the principles of neurorehabilitation: mass practice (repetitive training), high dosing (intensive training), structured practice, task-specific practice (ADL-relevant skills training), variable practice, multisensory stimulation (training in which the feedback is not limited to the visual modality), increasing difficulty (individualized training), explicit feedback (training that provides knowledge about the results), implicit feedback (training that provides task-relevant implicit signals), avatar representation (immersive training) and encouragement of the use of the paretic limb (training that counteracts compensation) [36].

In this sense, neurorehabilitation SVR systems allow rehabilitation work to proceed in a functional way and with specific intervention objectives, and these systems can easily evaluate and document progress during sessions [37]. Taking advantage of these characteristics, several authors have used virtual reality-based therapy (VRBT) to restore motor function after stroke [38–40]. Immersion, presence, and interactivity are three key features of virtual reality [41, 42]. In the course of our study, the exergames of the HandTutor^©^ glove software made it possible for the user to become the main character (immersion); users perceived the connection to the virtual environment through movement (interactivity) and acted inside it as they received input and responded to the challenges posed by the exergame (presence).

In this regard, Laver et al. [40] analyzed studies that compared N-SVR-based therapies with an alternative intervention or no intervention. In 2017, they updated their review by adding 35 new studies of N-SVR-based therapies, the majority of which used commercial games on the Nintendo Wii console. They concluded that virtual reality alone did not offer statistically significant improvements, in contrast to conventional treatment. However, when virtual reality was applied as a complement to common treatment, this combined treatment outperformed the conventional treatment alone. In these studies, the experimental group was given more time for treatment than the control group [41].

Choosing the appropriate neurorehabilitation strategies to maximize clinical results in stroke patients takes priority. In this sense, a combination of more traditional neurophysiological approaches and motion-based therapies, delivered at a high intensity and in a large dose in motivating game-related environments where motion can be made, offers an important advantage in restoring the motor function of the upper limb [29, 31].

Our clinical trial differs from the studies included in the review as follows: (1) it adds SVR technology (HandTutor^©^ glove), designed for hand motor rehabilitation; (2) it offers the same amount of time for intervention in both groups (control vs. experimental); and (3) it combines SVR with conventional treatment (experimental group). Additionally, many of the studies included in the review focused on adult patients with chronic stroke (a period of recovery equal to or greater than six months after diagnosis).

Ikbali and collaborators [39] used the Kinect sensor and the Xbox 360 console from Microsoft Inc.^©^ to train active movement of the upper limb, focusing on shoulder abduction and adduction and wrist flexion and extension exercises.

The Kinect sensor, independent of any specific software for rehabilitation after stroke, is able to capture gross movement of the upper limb, but it cannot identify hand motion and does not include exergames designed for hand motor rehabilitation.

Programs incorporating SVR technology to train distal motor function after cerebrovascular accident remain little known [43, 44], in contrast to programs focusing on proximal motor function [45], robot-assisted hand treatment [30, 46] or improving balance and walking [47, 48]. Therefore, the aim of the present study is to test whether conventional rehabilitation combined with SVR is more effective than conventional therapy alone in restoring the motor function and muscle tone of the hand after stroke.

It was hypothesized that, compared to control group (CG) participants, adults randomized to the experimental group (EG) would achieve an increased degree of hand motor function improvement and have superior results on the Fugl-Meyer Assessment, Ashworth Scale, and Action Research Arm Test.

## Methods

### Study design, randomization, and blinding

This was a prospective single-blind randomized controlled trial comparing conventional rehabilitation (CG) with the combination of conventional rehabilitation and an SVR system (EG); all reporting of this trial is in accordance with the Consolidated Standards of Reporting Trials (CONSORT) statement [49, 50].

The study was approved by the Research and Medical Ethics Committee of the Integrated [healthcare] Area of Talavera de la Reina (protocol code: 12/2018), and it complies with the Declaration of Helsinki. All participants received verbal and written information about the study and gave their written informed consent.

The participants were randomly assigned in a 1:1 ratio to the CG or the EG by a researcher who did not participate in the intervention or the evaluation process. The clinical practitioners who applied the conventional intervention and those who administered the baseline, postintervention, and follow-up clinical assessments to the intervention groups (CG and EG) were blinded. The participants and the researcher who applied VRBT could not be blinded.

### Participants

The study included 46 patients (43 of whom completed the intervention period and follow-up evaluation), with a mean age of 63.1 years (SD: 12.8). Nineteen percent (n = 8) were women. The participants were recruited from the Neurology and Rehabilitation units of the Hospital General Universitario of Talavera de la Reina, and none of them suffered from moderate or severe cognitive impairment that prevented them from following verbal and visual instructions from the therapist or the SVR technology. All participants had been diagnosed with stroke and met the following inclusion criteria: (1) age between 18 and 85 years; (2) maximum time of six months since diagnosis; (3) upper-limb motor impairment (Fugl-Meyer Assessment, Ashworth Scale and ARAT); (4) dependency in ADLs (Stroke Impact Scale; version 3.0); (5) life expectancy greater than 6 months (no diagnosis of any life-threatening condition such as end-stage cancer); and (6) absence of any other serious and disabling pathology.

According to baseline tests for determining motor function loss in the upper limb after stroke, we included patients who showed (1) scarce or no reflex activity, (2) absence or limitation of voluntary movements in flexion and extension synergies, (3) limitations in shoulder flexion–extension and adduction-abduction and wrist flexion–extension and stabilization, (4) difficulty grasping and gripping with the most affected hand, and (5) trembling or dysmetria (Fugl-Meyer Assessment). Furthermore, most of the patients selected for the study (especially those who had been diagnosed more than 20 days prior) showed a slight or a substantial increase in their muscle tone (Ashworth Scale) and difficulties in pinching, gripping or handling objects and making larger-scale movements, for example, placing their hand behind their head (Action Research Arm Test). We never set minimum or maximum scores for the testing tools applied; instead, we focused on movement limitations and elements that might affect the individual’s functional independence after stroke.

Four exclusion criteria were defined: presence of another neurological diagnosis, severe hemineglect, psychiatric pathology, and signed revocation of informed consent [51, 52].

### Intervention

Each participant completed 15 treatment sessions lasting 150 min each. These sessions took place on five consecutive days per week over the course of three weeks. The patients assigned to the EG received conventional upper and lower limb strength and motor training (100 min/session, administered by the hospital's physiotherapy and occupational therapy team) as well as rehabilitation with SVR devices (50 min/session), while participants in the CG received only conventional training in the form of physiotherapy (75 min/session) and occupational therapy (75 min/session). A 15-min break was allocated for each participant between professionals or types of treatment.

The conventional intervention protocol consisted of manual therapy techniques (massage); passive and active assisted mobilization of the upper and lower limbs; walking on level surfaces, slopes and stairs; exercises with resistance or assistance from balls, elastic bands and dumbbells in therapeutic cages and trellises; active assisted mobility exercises of the upper limb and fingers in a sitting position; moving objects horizontally on a table; elevation and superposition of objects in the vertical plane; and biomechanical tasks that simulated flexion–extension and abduction–adduction of the shoulder and flexion–extension of the wrist and fingers [51, 52].

The motor training protocol with SVR devices consisted of applying the HandTutor^©^ glove [53, 54], 3DTutor^©^ and Rehametrics^©^ [55]. All systems are based on intensive and repetitive practice through movement instructions, with feedback provided by the software and virtual environments and tasks that simulate the movements that the stroke survivor must complete in daily life [56, 57].

In this work, we will address the clinical and functional effects of the application of Meditouch^©^ software and the HandTutor^©^ glove.

The HandTutor^©^ glove focuses on flexion–extension of the fingers and wrist. It relies on analytical exergames that work isolated movements necessary to complete an ADL using virtual, gamified environments. It monitors and captures the movement of the hand joints (distal and proximal metacarpophalangeal and interphalangeal) and the wrist through sensors located in the front and back of the glove.

During the exercise, it provides visual and audio feedback on success and failure, shows the score and allows the therapist to modify the sensitivity of the movement to avoid frustration in the participant and reduce difficulty.

Meditouch^©^ software provides quantitative data on the progress of the patient in each exergame over the various treatment sessions. In addition, it generates a model with the silhouette of the hands, which allows the observation of active movement (shown in red) and passive movement (shown in blue) for each finger and the wrist. The exergames were customized according to the functional capacity of the patient.

**Fig. 1:**
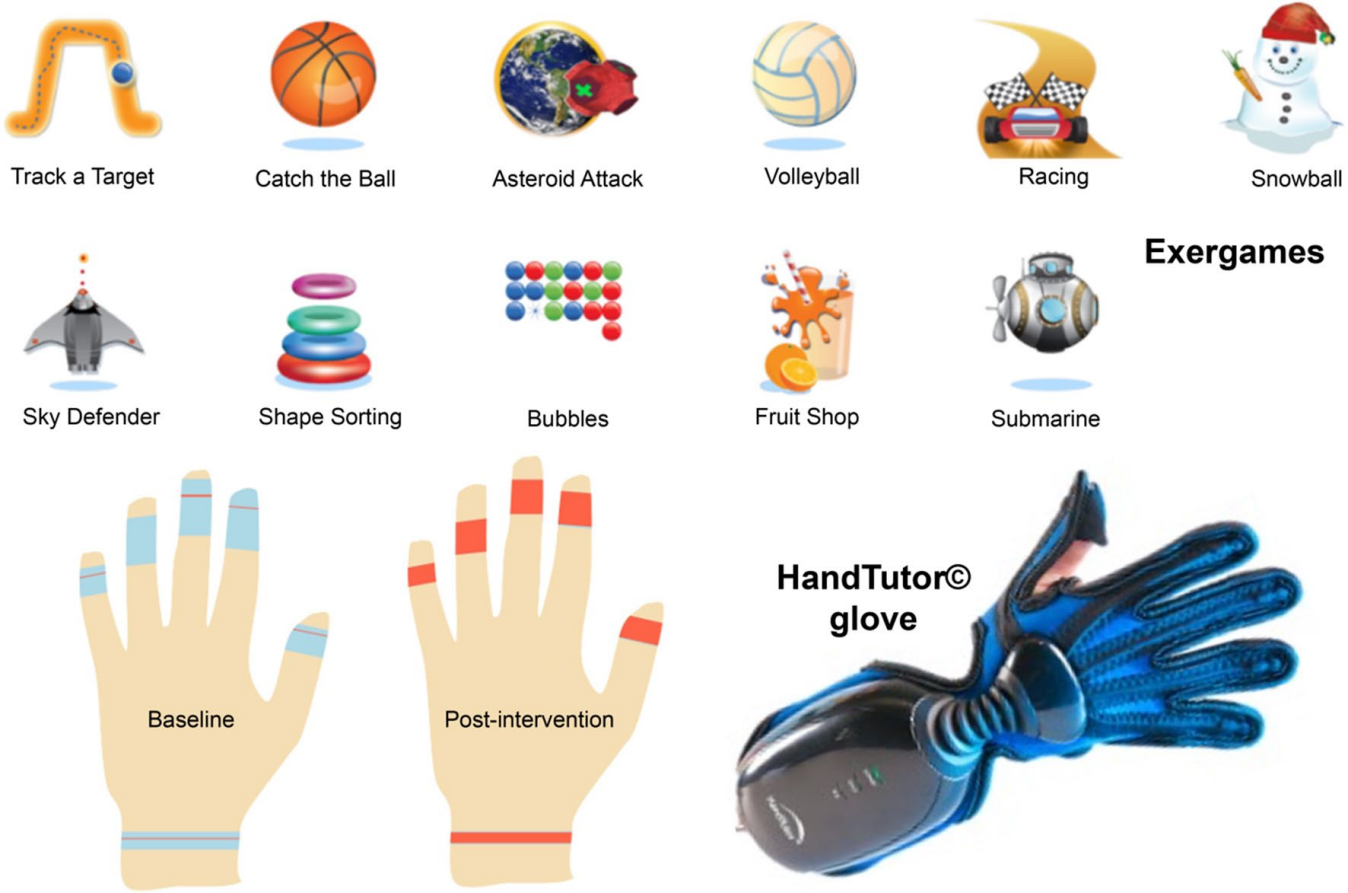
The SVR system (HandTutor ^©^ glove) used in this study, together with a representation of the active (red) and passive hand movement (blue) of a participant and the variety of exergames offered by the Meditouch^©^ software

Figure 1 shows the SVR system (HandTutor^©^ glove) used in this study, along with a representation of the active and passive movement of a participant’s hand and the variety of exergames offered by the software Meditouch^©^.

Although neither the clinical nor the functional effects of 3DTutor^©^ and Rehametrics^©^ are specifically analyzed in this work, a brief characterization of them is included, since they are among the systems applied in motor training protocols with SVR devices. The 3DTutor^©^ apparatus is a wireless device that monitors patients’ movements and includes evaluation of the full rotation of the joints in every plane, as well as choices for designing the treatment through the software exergames screen. The device can be placed on the distal or proximal side of any joint and is secured with Velcro strips of diverse sizes. Rehametrics^©^ uses the Kinect sensor by Microsoft^©^ to capture and monitor the movement of joints and body segments in real time. It works the upper limb (shoulder and elbow), trunk and lower limbs and simulates ADLs and community ambulation by means of virtual environments and in combination with games [52].

### Outcomes

In our study, we used the basic set of assessment measures recommended for clinical practice and research to assess the physical function and activity level of the upper limb; these measures are included in the current standardized protocols of evidence-based practice [58, 59].

The primary outcome was hand motor function. For quantification purposes, we applied the Fugl-Meyer Assessment-Upper Extremity (FMA-UE), the ARAT and the Ashworth Scale (a muscle tone evaluation).

Upper limb motor function (FMA-UE and ARAT) and muscle tone (Ashworth Scale) were evaluated and recorded before the treatment began (baseline), three weeks later (postintervention) and three months after its completion (follow-up).

### Fugl-Meyer assessment-upper extremity (FMA-UE)

The FMA is considered a valid, fully suitable instrument to evaluate the motor function of the upper limb after stroke, given its excellent psychometric properties and its appropriate scale. The upper-limb subscale of this instrument, the FMA-UE, is of great value for predicting the degree of independence that can be achieved one year after stroke. The FMA-UE is composed of 113 items, and each item on the evaluation scale is scored as an ordinal variable ranging from 0 to 2 points [60, 61]. We used the Spanish version of the FMA-UE [60], which has a Spearman coefficient of 0.946 (p = 0.000), excellent reliability (ICC 0.987; p = 0.000) and a Cronbach's alpha coefficient of 0.98 for motor control of the upper limb.

### Action research arm test (ARAT)

The ARAT assesses the ability to manipulate small and large objects, with a variety of qualitative items that allow a numerical quantification of each of the subtests: grasp, grip, pinch and gross movement [62]. Each item is scored on a 4-point scale, and total scores range from 0 to 57 points [63]. Decades ago, the interobserver reliability of this test in patients with poststroke hemiparesis was established to be 0.98, and the test–retest reliability was established to be 0.99 [64].

### Ashworth Scale

The Ashworth Scale measures resistance to passive movement on a scale of 0 to 4; it has a Kendall’s W of 0.765 (p = 0.000) for the elbow, and its reliability is 0.4 to 0.75 for 95% of the assessments [65].

### Data analysis

The sample size was calculated using the program Epidat 4.2. At the time when the study was designed, the methodologist and statistician calculated the size of the sample using an estimated effectiveness ratio of 90% in the experimental group and 50% in the control group, a power level of 80%, and confidence level of 95% (p < 0.05). The minimum necessary sample size was calculated to be 20 participants per group. Then, we decided to increase the sample size by 15% to compensate for potential losses (during field work and in the course of the study). The small sample size in each group could have led to a change in the variance in the case of missing data imputation, which would have compromised the accuracy of the final data.

Once we had gathered all the data, we carried out an analysis of missing values with SPSS; we observed a value below 5%, which can be attributed to chance. The estimated effectiveness in both groups (90% and 50%) was determined after considering the outcomes from studies already published on conventional poststroke motor rehabilitation, VRBT and the two approaches combined [43, 66, 67].

Student's t tests and Chi-squared tests were performed to compare the clinical and sociodemographic variables of the intervention groups. Differences in the Ashworth, ARAT, and FMA-UE scores at baseline, postintervention, and 3-month follow-up were analyzed with inter- and intragroup ANOVAs and Student's t tests. Statistical significance was defined by a p value of less than 0.05.

In this study of experimental intervention with the HandTutor^©^ SVR glove and Meditouch^©^ software, the outcomes of interest included the total score for upper-limb motor function according to the FMA-UE; the scores for the wrist and hand, which are important because they capture the clinical evolution of mobility in these specific body segments; and the differences found between the two groups receiving different interventions (EG vs. CG). We chose to include segment-specific scores because the technology we used to facilitate distal motor recovery of the upper limb (HandTutor^©^ glove) focuses on intensive rehabilitation of the wrist and the hand.

The analysis of missing data from the CG was carried out with multiple imputation in the analysis (expectation maximization and regression method), with a Little’s Chi-squared statistic of 36.280 (degrees of freedom = 28; p = 0.136).

The assignment of participants to the intervention groups was random and unknown to the researcher performing the statistical analysis.

## Results

### Characteristics of the participants

**Fig. 2:**
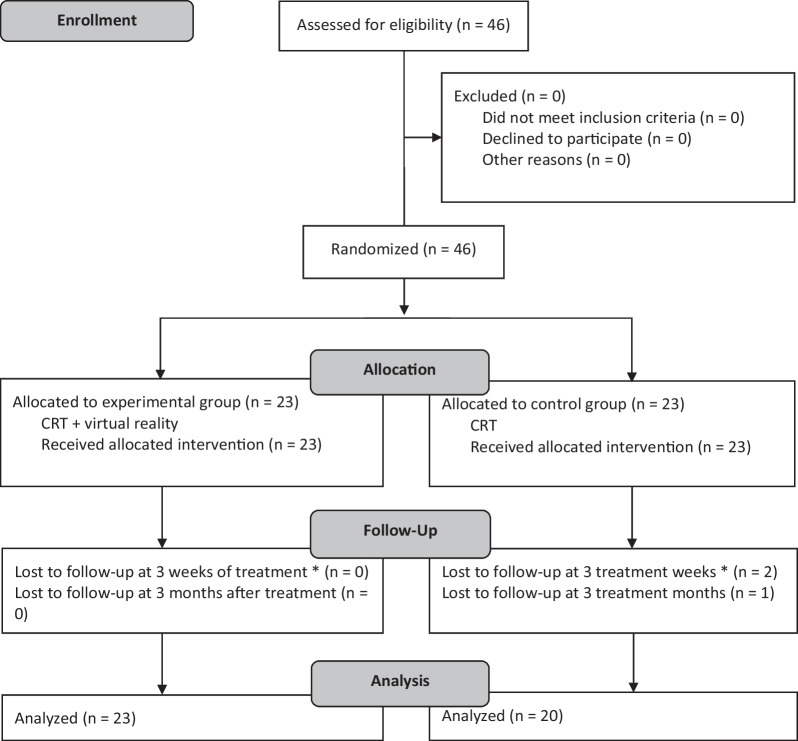
CONSORT flow diagram for participant recruitment, allocation, follow-up and analysis. *CRT* conventional rehabilitation treatment. *Postintervention evaluation

Twenty-three participants were assigned to the EG, and an equal number were assigned to the CG. Three participants in the CG were lost to follow-up due to the start of the COVID-19 pandemic in Spain (Fig. 2) [51, 52].

The characteristics of the participants are shown in Table 1. Significant differences were observed in the evolution of pain between the two groups, with pain decreasing considerably after intervention in the EG. Fifteen percent (n = 3) of participants in the CG reported a change in dominance (from right to left) during the first follow-up (postintervention), while the EG maintained the same hand dominance they had had at baseline [51, 52]. Regarding the results obtained from the visual analog scale of the EuroQoL instrument (EQ-VAS) for the measurement of self-perceived HRQoL, a statistically significant intergroup difference emerged after the experimental intervention (86.5 in the EG vs. 57.0 in the CG) [51]. Voluntary movement of the upper limb increased remarkably after the experimental intervention (FMA-UE: 30.1 in the EG vs. 24.7 in the CG; difference in means: 5.4), and, accordingly, muscle tone in the shoulder decreased (Ashworth Scale: baseline 1.30/postintervention 0.60 in the EG vs. baseline 1.22/postintervention 1.05 in the CG) [52].

**Table 1:** Characteristics of the participants in both groups (n = 43)

Characteristics: study variables	Experimental group (EG) (n = 23)	Control group (CG) (n = 20)	Difference in means between groups (p value)
Age			
Mean (SD) Under 55 years (%) 55 to 70 years (%) Over 70 years (%)	62.6 (13.5) 26 30 44	63.6 (12.2) 25 45 30	– 0.9 (0.812) 0.566
Sex (%)			
Male Female	78 22	85 15	0.571
Main diagnosis (%)			
Ischemic/thrombotic Hemorrhagic	91 9	90 10	0.883
Middle cerebral artery lesion (%)	61	55	0.697
Location of the brain injury (%)			
Right Left	83 17	85 15	0.832
Time since diagnosis (days)^a^			
Baseline (preintervention) Postintervention (3-week follow-up) Follow-up (3 months)	55.3 (34.3) 75.3 (34.3) 162.3 (36.9)	54.2 (30.4) 74.2 (30.4) 157.2 (36.1)	1.1 (0.909) 1.1 (0.909) 5.1 (0.650)

^a^Mean (SD)

p values were calculated using Student’s independent-samples t test for continuous variables and Pearson’s Chi-squared test for categorical variables

### Research objective

Table 2 shows the differences in the evolution of the Ashworth scores of the two intervention groups. A decrease in wrist muscle tone was observed in both groups, with a notably larger decrease in the EG (baseline/postintervention mean: 1.22 to 0.39). During follow-up, the muscle tone of participants in the EG increased slightly, while participants in the CG had a more pronounced increase over most of the range of wrist movement (postintervention/follow-up mean: 1.10 to 1.30). The effect size of the experimental intervention was 0.206 (difference between baseline and follow-up in the EG: 0.78; 95% CI 0.38–1.18).

**Table 2:** Linear model. Effect of the intervention on Ashworth Scale scores (wrist)

Intervention group				Difference between baseline and follow-up			
	Baseline mean (SD)	Postintervention mean (SD)	Follow-up mean (SD)	Mean (95% CI)	ANOVA		
					F	*p*	Partial η^2^
Ashworth Scale (wrist) (T score)							
Experimental group	1.22 (0.74)	0.39 (0.50)	0.43 (0.59)	0.78 (0.38/1.18)*			
Control group	1.13 (0.81)	1.10 (0.44)	1.30 (0.66)	− 0.10 (− 0.64/0.44)	10.6	0.002	0.206
p	0.706	0.000	0.000				

*SD* standard deviation, *95% CI* 95% confidence interval. Partial η^2^: effect size

*The difference in means is significant at p = 0.000

Table 3 shows the differences in the evolution of FMA-UE and ARAT results by intervention group. On the FMA-UE, we observed a score increase in both groups, but the change was notably larger in the EG (total motor function mean, baseline/postintervention: from 23.3 to 57.7; total wrist mean, baseline/postintervention: 3.4 to 9.0; total hand mean, baseline/postintervention: 3.9 to 12.8). During follow-up, the effects of the intervention in both groups remained stable, and the motor function of the upper limb increased slightly. The effect size of the experimental intervention was large (total motor function: 0.300; total wrist: 0.290; total hand: 0.299). On the ARAT, the EG quadrupled its score after the combined intervention, while the CG achieved approximately 50% of the total possible score for this test in the postintervention evaluation (maximum 57 points). The effect size of the experimental intervention was large (η^2^ = 0.321).

**Table 3:** Linear model. Effect of the intervention on FMA-UE (Total Motor Function, Wrist and Hand) and ARAT scores

Intervention group				Difference between baseline and follow-up			
	Baseline mean (SD)	Postintervention mean (SD)	Follow-up mean (SD)	Mean (CI95%)	ANOVA		
					F	p	Partial η^2^
FMA-UE (Total Motor Function) (T score)							
Experimental group	23.3 (6.9)	57.7 (4.7)	58.8 (5.9)	− 35.5 (− 38.9/− 32.0)*			
Control group	22.7 (5.4)	47.0 (6.1)	49.3 (6.3)	− 26.6 (− 29.1/− 24.2)*	17.5	0.000	0.300
p	0.722	0.000	0.000				
FMA-UE (Total Wrist) (T score)							
Experimental group	3.4 (1.9)	9.0 (1.0)	9.0 (1.0)	− 5.6 (− 6.4/− 4.8)*			
Control group	3.2 (1.7)	6.8 (1.2)	6.9 (1.3)	− 3.7 (− 4.5/− 2.8)*	16.7	0.000	0.290
p	0.688	0.000	0.000				
FMA-UE (Total Hand) (T score)							
Experimental group	3.9 (2.4)	12.8 (1.0)	12.8 (1.0)	− 8.9 (− 10.1/− 7.6)*			
Control group	3.5 (1.9)	10.0 (2.2)	10.0 (2.2)	− 6.3 (− 7.6/− 5.0)*	17.9	0.000	0.299
p	0.547	0.000	0.000				
ARAT (T score)							
Experimental group	13.2 (11.7)	46.0 (9.0)	46.0 (9.0)	− 32.8 (− 40.1/− 25.5)*			
Control group	11.5 (10.6)	29.3 (10.5)	29.7 (10.6)	− 17.4 (− 24.7/− 10.0)*	19.3	0.000	0.321
p	0.609	0.000	0.000				

*SD* standard deviation, *95% CI* 95% confidence interval. Partial η^2^: effect size

*The difference in means is significant at p = 0.000

## Discussion

The main purpose of this clinical trial was to test whether conventional rehabilitation combined with SVR technology is more effective than an intervention based exclusively on occupational therapy and physiotherapy for improving hand motor function in adults with subacute stroke.

Previous studies [40, 56] concluded that non-immersive virtual reality–assisted therapy using low-cost commercial platforms designed for family entertainment appeared to slightly contribute to the improvement of upper limb motor function after stroke. In 2017, these commercial games were compared with SVR technology to determine their efficacy against an alternative intervention or no intervention. None of the fifteen included studies focused on the combined use of SVR and conventional rehabilitation to improve hand motor function and normalize muscle tone of the hand in adults with subacute stroke [41].

In this study of patients with a poststroke interval of less than 6 months (EG mean: 55.3 days at baseline), patients randomly allocated to the EG followed a motor training protocol with SVR technology designed for improving hand motor function; interventions were delivered over the course of three weeks, five consecutive days a week, with each session lasting 50 min. In addition, patients in this group a received 100-min session of conventional treatment (occupational therapy and physiotherapy) along with each SVR training session. This combined treatment regimen yielded promising functional results regarding distal motor control of the upper limb, as resistance to movement decreased (spasticity), while the functionality and motion of the hand and wrist increased. In light of the functional results, it is important to highlight that the HandTutor^©^ glove is an affordable electronic device for rehabilitation teams and that it is versatile thanks to its small size, which allows it to fit in every clinical environment (hospital and healthcare centers); in contrast, expensive technologies such as robotics require not only a large economic investment but also facilities of specific dimensions.

The introduction of SVR systems into conventional rehabilitation processes offers new models of simulation that provide participants with the experience of motivating, stimulating, realistic environments, allowing safe practice of techniques based on meaningful, entertaining daily tasks [39].

Ikbali et al., in their intervention, chose the Kinect sensor in combination with the commercial console Xbox 360 by Microsoft^©^ Inc. for training active movements of the upper limb (shoulder abduction and adduction and active wrist flexion and extension); their team concluded that game-based therapy contributes to motor and functional recovery of the upper limbs when administered in the subacute phase as a complement to conventional rehabilitation for patients diagnosed with stroke [39]. However, few studies have introduced SVR technology into hand treatment with software specifically designed for clinical use in patients with neurological disorders, as we do in the course of our work.

The results of this study suggest that VRBT using Meditouch^©^ software and HandTutor^©^ gloves in combination with conventional physiotherapy and occupational therapy is effective in the rehabilitation of motor function in the subacute phase of stroke.

The differences in Ashworth Scale scores for the wrist found in our study coincide with the results published by Young-Bin et al. [68]. In contrast to the present work, their study combined the SVR intervention with the use of real objects, prolonged the intervention to six weeks, and delayed the follow-up evaluation to 4 weeks. The matching findings may indicate that the effect of experimental intervention on decreased wrist muscle tone is maintained over time. In our case, we were able to check only the baseline/postintervention change after three weeks of combined treatment (SVR and conventional physiotherapy and occupational therapy treatment) and the progress after three months of follow-up (baseline/follow-up difference: 0.78 points; p = 0.000; effect size = 0.206).

Our study revealed positive results in terms of hand function after the combination of SVR and conventional physiotherapy and occupational therapy. The difference between baseline and follow-up measurements reflected functional improvements after the experimental intervention: 5.6 points on the wrist scale of the FMA-UE, 8.9 points on the hand scale of the same instrument (p = 0.000 for each), and 32.8 points on the ARAT. In this respect, it is important to note the proximity of the three-week postintervention differences to the minimum detectable change on the Spanish version of the FMA-UE, which corresponds to 7.3 points six weeks after intervention. The effect size of the intervention (h^2^) for the wrist and hand were 0.290 and 0.299, respectively). Factors that may have contributed to the large improvement in upper-extremity motor function include the repetitive and intensive practice involved in the experimental intervention and the use of a structured training approach whose objectives were focused on functional motor training. In poststroke SVR treatment, special attention was paid to the restoration of the different biomechanical movements and curvature of the hand to provide a stable base and correct alignment. The interface used by the therapist to adjust the difficulty levels of the exergame and to adapt to each participant’s ability and progression in the EG may also help explain the results. The training objectives focused on primary motor components, restoring selective voluntary movements to reach and grasp objects in the virtual environment. It is important to note the characteristics of the participants, who had a poststroke interval of less than 56 days (SD between 30 and 34) and a mean age of 63.1 years ± 12.3. Participants in this study did not have elevated levels of muscle tone at baseline measurement, and those with severe hemineglect (which implies a negative poststroke prognosis) were excluded.

Increased practice results in greater ability as long as the exercises are challenging, progressive and skill-based [69]. In this regard, our results showed that, from the eighth combined treatment session with SVR technology, patients displayed clinical improvements in the fine motor skills and functionality of the hand, which progressively increased until the end of experimental treatment. In this study, the experimental design called for three consecutive weeks of combined treatment (EG) with SVR sessions of 50 min each. However, a larger study with varying durations and session lengths is needed to determine the optimal intervention protocol.

A noteworthy feature that likely contributes to the utility of the HandTutor^©^ glove’s exergames is that they offer progressive challenge levels, allowing therapists to increase the difficulty, the frequency of stimuli, and the presence of distractors according to the evolution of motion and functionality in each individual stroke patient.

For our study, we designed a daily intervention plan, devoting 50 min per session to motor training with SVR technology devices.

High-quality upper-limb training focusing on the motion quality of the injured segment is difficult to achieve in the time allowed in standard rehabilitation programs, in which patients are encouraged to carry out tasks through compensation [70].

Similar results have been found in studies that combined conventional techniques with the use of robotics [29, 31]. In these cases, the robotic system was applied in fewer weekly sessions than our SVR system. It is possible that the effect of robot-assisted intervention is more durable than that of SVR technology. Predictive models, as yet unpublished, are needed to help identify the weekly dose that yields optimal poststroke recovery.

The use of VRBT in treatments aimed at functional recovery of the upper limb in stroke patients brings about an enriched, interactive environment that positively influences neuroplasticity, especially in the subacute phase of the disease, in which the level of brain reorganization is at a maximum [71]. No significant differences were found in the baseline evaluations of the intervention groups; therefore, we can conclude that the results are a consequence of the use of SVR technology in active and intensive training and the response to the demands and challenges of exergames [72]. The use of SVR as a rehabilitation method formulates an alternative therapeutic concept that is attractive to patients, as it allows them to focus on the demands of the exergames and not on the need for repetition or the degree of challenge. This approach facilitates the relearning of coordinated motor patterns and enables environments to be created in which the intensity of visual and auditory feedback and training can be systematically manipulated and enhanced to create individualized motor learning paradigms and increase treatment compliance, thus positively affecting the emotional state of patients [73].

The above results suggest that SVR-facilitated exercise-based intervention programs benefit the recovery of hand motor function in people with stroke.

Recent studies on stroke patients observed significant changes and great improvements in ARAT and FMA-UE results when patients received upper-limb therapy 5 days a week over 3 , 6 and 12 weeks; the declared intention in those studies was to make motion practice as realistic as possible, with increasing progress in joint movement culminating in full practice of the actual physical task [74–76].

The SVR technology devices used for our study focused on the joints and body segments damaged by stroke, differentiating and isolating the movements and the active range of joint motion if necessary. The HandTutor^©^ glove enables the therapist to block and/or reduce the range of motion if compensation is noted in the wrist during flexion and extension of the fingers. In addition, the software makes it possible to isolate the fingers if the patient always reaches the goal with the same movement and body segment. In this way, the digital and tripod grasps (necessary for carrying out ADLs) can be isolated and trained separately.

Evidence from this study reveals that virtual reality has the potential to create effective environments for rehabilitation with safe, multimodal, individualized simulations while providing feedback and options regarding repetition, intensity, and training in specific tasks; these features are not common among conventional physical therapies but are useful for promoting the recovery of upper limbs after stroke [33, 77–79].

## Limitations of the study

The results of the present study cannot be extrapolated to other poststroke phases or to rehabilitation programs with fewer weekly sessions.

The design of our study does not include a long-term follow-up to clarify the evolution of the intervention groups (CG vs. EG) or the differences found. In addition, this study was limited to a single center, in contrast to several studies with multicenter randomized controlled designs [51, 52, 80]. The psychometric properties of the items described for the upper limbs (total wrist and total hand) are unknown, and the participants could not be blinded because they agreed not only to receive conventional rehabilitation treatment but also to report the course of changes in their mobility and execution of ADLs as a result of SVR-based rehabilitation. In future research, it would be interesting to extend the follow-up period to 12 months to ascertain whether the effect of the experimental intervention is maintained in the chronic poststroke phase. It would also be advantageous to increase the sample of participants with a less intensive conventional intervention group (two or three days a week); this would allow blinding of the participants.

## Conclusions

The outcomes of the study suggest that conventional rehabilitation combined with an SVR-based method can be more effective than conventional therapy alone in improving hand motor function and voluntary movement and normalizing muscle tone in subacute stroke patients. With combined treatment, hand and wrist functionality and motion increased; resistance to movement (spasticity) decreased, and this decrease was sustained over time.

Patients showed significant clinical improvements in the fine motor skills and functionality of the affected hand, with a large effect size, and these gains were achieved in less time than if conventional rehabilitation alone were used.

## Data Availability

Not applicable.

## Abbreviations

ADLs: Activities of daily living
HRQoL: Health-related quality of life
N-SVR: Nonspecific virtual reality
SVR: Specific virtual reality
VRBT: Virtual reality-based therapy
CG: Control group
EG: Experimental group
CONSORT: Consolidated Standards of Reporting Trials
SD: Standard deviation
ROM: Range of motion
FMA-UE: Fugl-Meyer Assessment-Upper Extremity
ARAT: Action Research Arm Test
SIS: Stroke Impact Scale
CI: Confidence interval
